# Flow Cytometry for Rapid Enumeration and Biomass Quantification of Thraustochytrids in Coastal Seawaters

**DOI:** 10.1264/jsme2.ME17162

**Published:** 2018-06-16

**Authors:** Yingbo Duan, Biswarup Sen, Ningdong Xie, James S. Paterson, Zixi Chen, Guangyi Wang

**Affiliations:** 1 Center for Marine Environmental Ecology, School of Environmental Science and Engineering, Tianjin University Tianjin 300072 China; 2 School of Biological Sciences, Flinders University GPO Box 2100, Adelaide SA 5001 Australia; 3 Laboratory of Synthetic Microbiology, School of Chemical Engineering & Technology, Tianjin University Tianjin 300072 P. R. China

**Keywords:** thraustochytrids, flow cytometry, abundance, biomass, bacterioplankton

## Abstract

Marine fungus-like eukaryotic unicellular protists (thraustochytrids) are considered to play an important role in the marine microbial food web. However, their abundance, distribution, and relative biomass in coastal waters have not yet been examined in detail. By using a flow cytometry method (FCM) for the rapid enumeration of thraustochytrids in nearshore and offshore stations along the Gulf of Bohai, China, we herein expanded current knowledge on their ecological significance. The FCM method allows for the rapid detection and quantification of prokaryotic and eukaryotic cells, but is rarely applied to the enumeration of small eukaryotic protists. Epifluorescence microscopy (EpiM) has been commonly used for the direct detection and enumeration of thraustochytrids; however, this method is time-consuming and inapplicable to a large-scale analysis of complex seawater samples. There is no available FCM method to track the abundance and biomass of thraustochytrids in marine habitats. The FCM enumeration of thraustochytrids in seawater samples ranged between 400 and 4,080 cells mL^−1^ with a biomass range of 8.15–83.96 μg C L^−1^. The thraustochytrid biomass contributed 10.9% to 98.1% of the total biomass of the heterotrophic microbial community comprising bacterioplankton and thraustochytrids. Their overall abundance in nearshore stations was significantly different from that in offshore stations (*P*<0.5). The present results provide an optimized method for the rapid detection and enumeration of thraustochytrids in seawater and facilitate large-scale studies of the ecological role of thraustochytrids in the microbial food web of coastal waters.

Thraustochytrids (~2.0 to 20.0 μm) are marine, unicellular, fungus-like marine protists (32) that are characterized by the presence of a sagenogenetosome, ectoplasmic net, and cell wall composed of non-cellulosic scales (27). They are ubiquitous in seawater, sediments, algae, and invertebrates, both as saprotrophs and parasites (32). Since they are similar to bacterioplankton in their osmo-heterotrophic mode of nutrition, they are considered to play a role in the remineralization of particulate and dissolved organic matter, similar to bacterioplankton, and form an important link in the food web (20, 32). They are alternative food sources for picoplankton feeders and active degraders and consumers in marine microbial food chains (24) with a dual role in the breakdown of complex organic molecules and bacterivory (28). Thraustochytrids also have promising biotechnological applications because of their capacity to produce polyunsaturated fatty acids (PUFAs) (*e.g.*, DHA & EPA), enzymes, polysaccharides, and secondary metabolites (20, 33, 34, 40, 41).

In order to clarify the ecological function of thraustochytrids in marine ecosystems, it is critical to obtain accurate quantitative data on their abundance and biomass. Epifluorescence microscopy (EpiM) has been commonly used for the direct detection and enumeration of thraustochytrids using an acriflavine staining technique (29). EpiM has been successfully used to estimate the abundance of thraustochytrids in different habitats (12, 13, 25, 38) and demonstrated that coastal waters may attain a few hundred thousand cells per liter of seawater and occasionally attain biomass up to 50% of bacterioplankton (4). Although EpiM allows for the realization of thraustochytrid density and biomass in seawater, it may be time-consuming due to the complexity of natural seawater, particularly when a large number of cell counts are necessary within a short period of time for a large-scale study (6). In addition, fixation artifacts, misidentification, human error, and over- or under-staining are other sources of error that severely limit EpiM (37). Therefore, the lack of a rapid and reliable tool for the enumeration of thraustochytrids in seawater has restricted the scope of studying the abundance and biomass of this important group of small heterotrophic protists.

In recent years, the rapid detection and enumeration of microbes have been one of the major objectives of marine biologists (47). Flow cytometry (FCM) is a well-established method for microbial analyses at the community and single-cell levels in the field of aquatic microbiology (48). As a useful tool for the enumeration of picoplanktonic cells, FCM has been successfully applied to phytoplankton, bacterioplankton, and protists in oceanic samples (5, 14, 43, 44, 47, 50). Heterotrophic protists in cultures have been successfully enumerated using FCM (37). However, a FCM analysis of these protists is often regarded as a niche area with a handful of applications. These protists are extremely diverse, incorporating single-cell and multicellular forms. Thus, different strategies for staining and cell sorting need to be adopted for the different species of heterotrophic protists and their various physiological states. Many methodologies and staining techniques have been developed principally for studies on planktonic protists. Nevertheless, there is currently no available staining technique for the FCM detection of fungus-like protists—thraustochytrids.

Mostly coastal waters are featured with high biodiversity and high primary production. Due to excessive primary production, a large fraction of primary organic matter becomes available as detritus to thraustochytrids, bacterioplankton, and other planktonic microorganisms (39, 40). Bacterioplankton have long been known to play a key role in the degradation of this detritus, and in the export and storage of organic matter in coastal ecosystems (35). Thraustochytrids are considered to play a role in the remineralization of organic matter similar to bacterioplankton; however, these heterotrophic protists may occupy a distinct ecological niche in the sea in order to avoid competition from ubiquitous bacterioplankton (4). The mechanisms by which thraustochytrids compete with bacterioplankton in coastal waters are an interesting subject of study. Therefore, empirical evidence integrating the abundance and distribution of thraustochytrids and bacterioplankton as well as the relative biomass patterns of these groups is crucial.

In the present study, we developed an FCM protocol for the rapid detection of thraustochytrids in natural seawater based on the staining principle of the EpiM method. We adapted the fluorescent dye acriflavine to stain the thraustochytrid cell wall (containing sulfated polysaccharides) red and the nucleus yellow-green (30). Photosynthetic protists were easily distinguished by their pigment autofluorescence, heterotrophic protists by the absence of cell wall fluorescence (30), and detritus with relatively low green fluorescence (FL1) and relatively high side scattering (SSC) (37). The FCM protocol developed was then applied to track spatiotemporal variations in the abundance and relative biomass fraction of thraustochytrids in the coastal seawaters of the Gulf of Bohai, China. This study broadens the scope of FCM applications in aquatic microbiology and is the first to report the detection and enumeration of marine fungus-like protists (thraustochytrids) in coastal seawaters.

## Materials and Methods

### Strains and cultures

Five previously isolated thraustochytrid strains (*Aurantiochytrium limacinum* PKU#SW7, *Aurantiochytrium* sp. PKU#Mn11, *Schizochytrium limacinum* PKU#Mn4, Thraustochytriidae sp. PKU#Mn16, and Thraustochytriidae sp. PKU#SW8) from coastal marine habitats were used to develop the FCM method (18). All strains were cultured in flasks containing Mn4 medium (2% [w/v] glucose, 0.025% [w/v] KH_2_PO_4_, 0.15% [w/v] peptone, 0.1% [w/v] yeast extract, pH 7.0) supplemented with 0.075% (w/v) streptomycin and 0.05% (w/v) ampicillin, and incubated at 30°C on a shaker at 150 rpm (11).

Axenic culture samples were individually prepared by sampling after 60 h of incubation and subsequent dilution (1:1,000) with 0.22-μm filtered TE buffer. In order to obtain a collective FCM signature of the five strains, a mixed culture sample (Mix) was prepared by mixing together the five axenic cultures (grown for 60 h) in equal proportions. In order to mimic natural seawater, which consists of different populations at various growth stages, we performed an analysis of mixed culture samples that were prepared through growth at various incubation times: 12 h (Mix-12h), 24 h (Mix-24h), 48 h (Mix-48h), and 60 h (Mix-60h). A final mixture (Pooled-Mix) was prepared by pooling all the samples of these incubation times to model natural seawater. Dilutions (1:1,000) of Mix and Pooled-Mix samples were performed with 0.22-μm (polycarbonate isopore membrane filter; Millipore, USA)-filtered and autoclaved natural seawater.

### Natural seawater samples

Sampling was conducted in May and July 2014 along three parallel sections (C, E, and F) at the coast of the Gulf of Bohai, China. The map of sampling stations is provided in Fig. S1. Along each section, two stations (nearshore and offshore) were sampled. Water samples for nearshore stations were collected at two depths, each from the surface (*ca.* 1 m) and bottom (*ca.* 7 m), while those for offshore stations were collected at three depths, each from the surface (*ca.* 1 m), subsurface (*ca.* 10 m), and bottom (*ca.* 21 m). Thirty seawater samples were analyzed. Water samples for the FCM analysis of thraustochytrids were transferred into 4-mL cryovials in triplicate, fixed with 0.22-μm filtered formaldehyde (2% [v/v] final concentration) (4), and then incubated at 4°C for 3 h (51). In the bacterioplankton FCM analysis (7), seawater samples were transferred into 2-mL cryovials in triplicate, fixed with 0.22-μm filtered glutaraldehyde (0.5% [v/v] final concentration), and incubated at 4°C for 15 min (8). All samples for the FCM analysis after the cell fixation step were stored at −80°C until further analyses (17, 27). Samples for the microscopic analysis of thraustochytrids were transferred into 50-mL centrifuge tubes in duplicate, fixed with 0.22-μm filtered formaldehyde (2% [v/v] final concentration) (4), and stored at 4°C until analyzed.

Environmental parameters were analyzed following the methods described in our previous study (10).

### Sample staining for FCM

Acriflavine (3,6-diamino-10-methylacridinium chloride mixture with 3,6-diaminoacridine) was used to concurrently stain thraustochytrid cell walls containing sulfated polysaccharides (red) and the nucleus (yellow-green), as described previously (30). Thraustochytrid cultures and seawater subsamples were stained by adding 12 μL acriflavine hydrochloride (Sigma, Germany) solution (5 mg mL^−1^ in TE buffer) into 3 mL of the sample. After a brief vortex, the resulting solutions were incubated in the dark at room temperature for 30 min. Seawater subsamples for the bacterioplankton analysis were diluted (1:10) with 0.22-μm filtered TE buffer and then stained with 12.5 μL SYBR-I Green solution (1:500 dilution; Molecular Probes, Eugene, OL, USA), followed by an incubation in the dark at room temperature for 10 min (8). Yellow-green fluorescent polystyrene latex beads with a diameter of 1 μm (Molecular Probes) were added to each FCM sample as an internal standard.

### FCM analysis

A FACSCalibur flow cytometer (BD-Biosciences, Franklin Lakes, NJ, USA) equipped with an air-cooled argon-ion laser (488 nm, 15 mW) was used to count thraustochytrids and bacterioplankton. BD FACS Flow Sheath Fluid was used as sheath fluid. All samples were acquired at a high flow rate (60 μL min^−1^) over an optimum time of 3 min to collect a sufficient number of cells. In the thraustochytrid analysis, SSC, forward scatter (FSC), FL1, and red fluorescence (FL3) were recorded for each sample. FL1 was used as the triggering parameter to measure signals from particles with FL1 intensity exceeding the threshold value only. CellQuest Pro software was configured to record particles with detectable FL1, and the threshold was raised until the event rate decreased to less than 1,000 events s^−1^—the upper limit of the processing speed of the software (37). The cell properties—FL1, FL3, SSC, and FSC—were acquired with log amplification and data analyzed with CellQuest Pro software (BD-Biosciences). A polygon gate was drawn around each population and the events in the polygon were then counted using CellQuest Pro software. Thraustochytrid cells inside the gate criteria were sorted and filtered onto a 25-mm Isopore hydrophilic membrane with a pore size of 0.2 μm (Merck Millipore) for a further analysis in order to ensure the identity of thraustochytrids under blue excitation light using the Eclipse N*i*-U (Nikon Instruments, Tokyo, Japan) epifluorescence microscope (Fig. S2).

In the bacterioplankton analysis, SSC, FSC, and FL1 were recorded for each sample. Samples were acquired at a high flow rate (60 μL min^−1^) for 2 min. In data acquisition, we followed the method described above for thraustochytrids. The bacterioplankton population in individual samples was detected and enumerated based on the cell SSC and SYBR FL1 (9, 16, 22, 23).

In each sample, a triplicate analysis and counting were performed for thraustochytrids and bacterioplankton. Total thraustochytrid and bacterioplankton counts were obtained by correcting the measured total counts for noise (lowest FL1) with TE buffer or sterile and 0.22-μm filtered seawater. In order to calculate the abundance of thraustochytrids and bacterioplankton from the number of events in a flow cytogram, a known concentration of beads (Molecular Probes) was added to each sample to serve as the internal standard. The beads were appropriately diluted to create a working stock that was enumerated at the start of each experiment using the Eclipse N*i*-U (Nikon Instruments) epifluorescence microscope.

### EpiM counts

The EpiM counts of thraustochytrids were taken as the reference standard to assess the FCM quantitation. Subsamples of the culture (5 mL) and natural seawater (25 mL) were filtered onto a 0.2-μm black polycarbonate isopore membrane filter (Millipore, Burlington, MA, USA). The resulting filter was stained with 4 mL of 0.05% (v/v) acriflavine in 0.1 M citric buffer (pH 3.0) for 4 min and excessive staining solution was removed using a vacuum. A total of 1 mL of 70% (v/v) isopropanol was added for differentiation and the filter was then rinsed with sterile distilled water (30). The filter was mounted on a microscope slide and observed under blue excitation light (488 nm) using the Eclipse N*i*-U (Nikon Instruments) epifluorescence microscope equipped with a 12V-100W LL Halogen Lamp. Twenty different microscopic fields were counted for each individual sample based on their characteristic red cell walls and yellow-green nuclei. Each sample was analyzed in duplicate (Table S1).

### Relative biomass assessment

The contribution of thraustochytrids over the total biomass of bacterioplankton and thraustochytrids was assessed in order to demonstrate their relative abundance in natural seawater samples. The biomass estimation was based on FCM counts. The bacterioplankton biomass was estimated based on a value of 30.2×10^−15^ g carbon (C) cell^−1^ (7). The thraustochytrid biomass was estimated based on a value of 20.6×10^−12^ g C cell^−1^ (12). The relative biomass fraction was calculated as the thraustochytrid or bacterioplankton biomass over the total biomass of bacterioplankton and thraustochytrids.

### Statistical analysis

The relationship between thraustochytrid numbers assessed by FCM and those by EpiM was analyzed by a least-square linear regression (Origin software 6.0).

Post-hoc tests according to Nemenyi tests for multiple comparisons of the (mean) rank sums of the FCM counts of samples across sections and shores were performed after a Kruskal-Wallis test in R (version 3.4.2). In order to analyze the relationship between thraustochytrids and environmental data, we performed a Principal Components Analysis (PCA) using Canoco 5 software and Pearson’s correlation test in IBM SPSS Statistics 22 on the dataset consisting of environmental variables and the abundance of bacteria and thraustochytrids in all samples. Data were centered and standardized prior to PCA.

## Results

### Validation of FCM enumeration

**Fig. 1:**
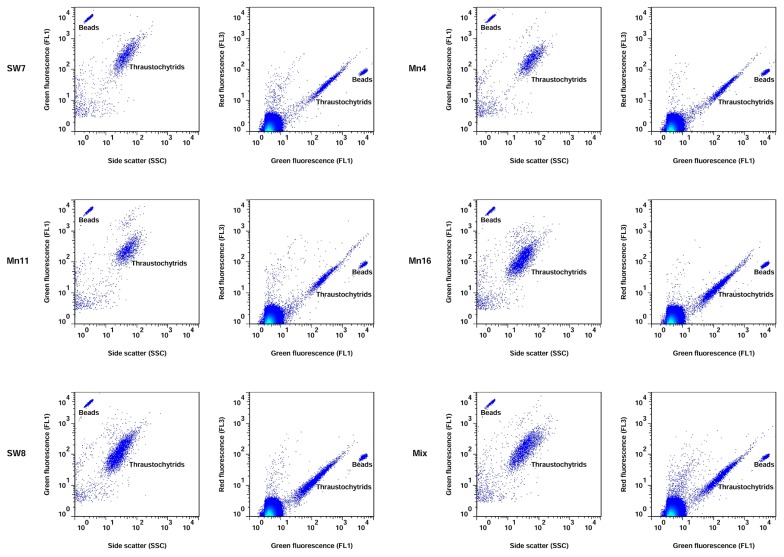
Flow cytometry signatures of axenic and mixed cultures of thraustochytrids. SW7 (*Aurantiochytrium limacinum*), Mn11 (*Aurantiochytrium* sp.), Mn4 (*Schizochytrium limacinum*), Mn16 (*Thraustochytriidae* sp.), and SW8 (*Thraustochytriidae* sp.) were grown for 60 h and then diluted with 0.22-μm filtered TE buffer. ‘Mix’ represents the mixture of SW7, Mn11, Mn16, Mn4, and SW8 diluted with 0.22-μm filtered TE buffer.

The five thraustochytrid axenic cultures and mixture (Mix) were detected and gated based on their FL1 (DNA-induced fluorescence) vs. FL3 (sulfated polysaccharide-induced fluorescence) and FL1 vs. SSC (size and granularity) signals (Fig. 1). The FL1, FL3, and SSC signals of the five axenic cultures and their mixture were uniform. The FL1 vs. FL3 cytograms of all cultures displayed nearly similar ratios of FL3 and FL1, confirming the simultaneous staining of their cell wall and nuclei. The FL1, FL3, and SSC signals of strains Mn16 and SW8 were slightly lower than those of the strains SW7, Mn11, and Mn4. Histogram plots (Fig. S3) of the cultures showed a peak shift for FL1 of Mn16 and SW8 from other strains; however, the peak area of the Mix sample was approximately an average of the peak areas of all the strains. Thus, the combination of five different thraustochytrids into one sample (Mix) provided identical results to the analysis of individual axenic cultures, demonstrating that the results obtained with individual cultures may be extrapolated to the thraustochytrid community in natural seawater. The EpiM examination of the sorted cells from natural seawater samples inside the FCM gate criteria confirmed that their morphology was similar to thraustochytrids (Fig. S2). This further ensured that the counts of the events only represented thraustochytrids and not any other particles. Overall, the FCM signatures of axenic cultures suggested a uniformity in the content of the cell wall and nucleus and the size of thraustochytrid cells across different strains.

**Fig. 2:**
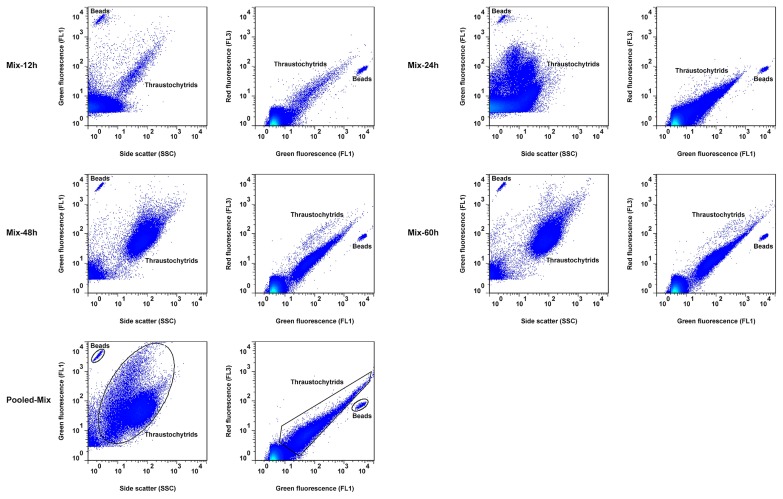
Detection and discrimination of thraustochytrid population growth over time. Each flow cytogram shows the distinct signature of the thraustochytrid culture mixture (Mix) at 12, 24, 48, 60, and 72 h of growth. ‘Mix’ is a mixture of the SW7, Mn11, Mn16, Mn4, and SW8 cultures at each incubation time diluted with 0.22-μm filtered seawater. ‘Pooled-Mix’ is a mixture of the Mix-12 h, Mix-24 h, Mix-48 h, and Mix-60 h samples mimicking natural seawater.

Cell cycle-dependent events and cell states in a microbial culture are related to the age of the culture (24, 37, 45). We herein studied cytograms of the mixed culture grown over various incubation periods in order to assess the power of FCM in displaying cell-cycle events and cellular characteristics. As shown in Fig. 2, the SSC of the Mix-24 h sample was markedly different from those of the Mix-12 h, Mix-48 h, and Mix-60 h samples. This result suggests the non-uniform cell size of thraustochytrids at 24 h of the growth phase resulting from different cellular contents during rapid cell proliferation. The FCM signature of the 24-h cytogram eventually appeared to reflect the exponential phase of the cell cycle. The relatively low number of events in the 12-h cytogram represented the phase of the cell cycle before the start of cell division. The uniform signal ratio of FL1 to SSC and FL1 to FL3 of the Mix-48 h and Mix-60 h samples appeared to represent the stationary cell cycle stage when cells have achieved growth maturity with similar sizes and contents. Thus, FCM allowed for the unequivocal differentiation of the mixed culture based on ongoing cell-cycle stages. The Pooled-Mix sample cytogram displayed a collective profile with values for the FL1, FL3, and SSC signal parameters that were approximately the average of all the values characterizing Mix samples at various growth stages. More importantly, the FL3-FL1 signature of the Pooled-Mix sample displayed a uniform region and was the best reference gate to detect and enumerate thraustochytrids in natural seawater samples.

In order to validate the reliability and accuracy of the FCM protocol, the ratios of FCM counts to microscopy counts (FCM/EpiM) of the axenic culture and Mix samples were evaluated. The range of FCM/EpiM counts was between 1.25 and 2.79. This indicated that the value of thraustochytrid counts derived from FCM was higher than that of EpiM. The average of the FCM counts of five axenic cultures was 6.2×10^7^ cells mL^−1^ and was close to the FCM count of 6.6×10^7^ cells mL^−1^ obtained from the Mix sample. However, the average EpiM count of 2.5×10^7^ cells mL^−1^ of five axenic cultures and that of 3.2×10^7^ cells mL^−1^ obtained for the Mix sample were markedly different.

### Thraustochytrid abundance in natural seawater

**Fig. 3:**
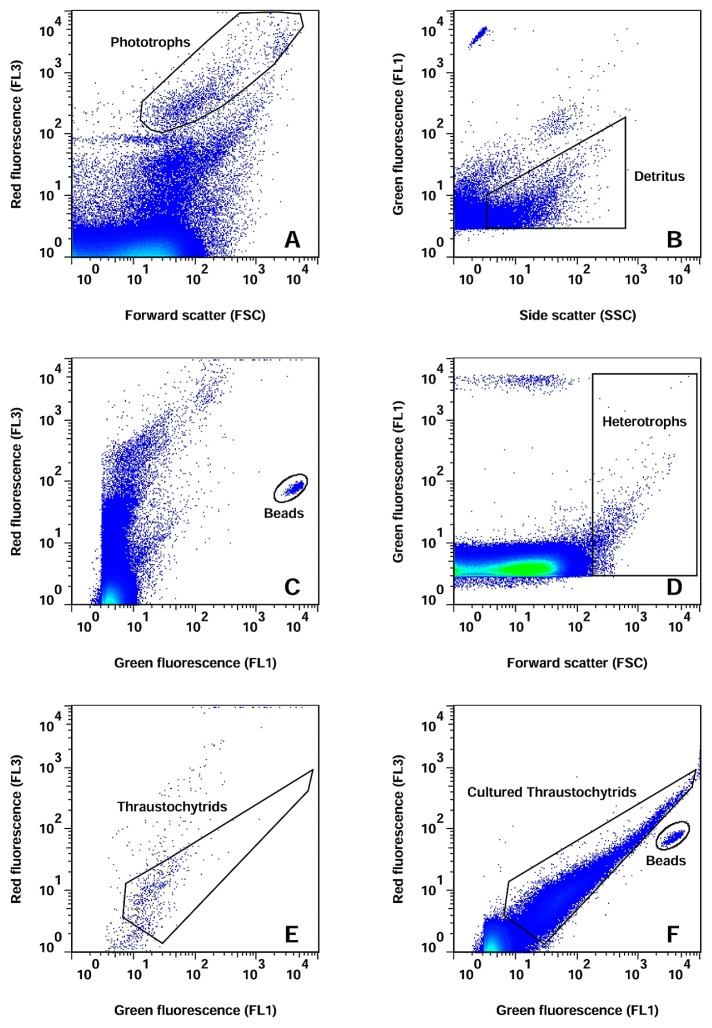
Detection of thraustochytrids in natural seawater samples with the developed flow cytometry protocol. (A) Phototrophs were discriminated based on relatively high chlorophyll fluorescence in a cytogram of red fluorescence (FL3) vs. forward scatter (FSC); (B) Detritus were distinguished based on relatively high side scatter (SSC) and relatively low green fluorescence (FL1) in a cytogram of FL1 vs. SSC; (C) Beads with a diameter of 1.0 μm were used for size estimation and gated based on their high FL1 and relatively low FL3 in a cytogram of FL3 vs. FL1; (D) Phototrophs and detrital particles were removed from the plot of FL1 vs. FSC, and the remaining events larger than 2.0 μm were gated as heterotrophic protists >2.0 μm based on the bead size reference; (E) Events of heterotrophic protists >2.0 μm were then plotted in a cytogram of FL1 vs. FL3, allowing for the better discrimination of thraustochytrids from other heterotrophic nanoplanktonic protists (F) The flow cytometry signature of thraustochytrids in culture samples (Pooled-Mix).

The abundance of thraustochytrids in natural seawater was measured by FCM based on a sequential analysis of a representative seawater sample. The sequential analysis involved the discrimination of each of the different cell populations and particles based on their light scatter and fluorescence data. Phototrophic eukaryotes were detected based on their relatively high chlorophyll fluorescence in the cytogram of FL3 vs. FSC (Fig. 3A)—displaying high FL3 relative to size. Detrital particles with a low FL1 were discriminated in the cytogram of FL1 vs. SSC (Fig. 3B). Beads with high FL1 were used to establish a minimum size for heterotrophs and were displayed on the cytogram of FL3 vs. FL1 (Fig. 3C). With beads as the size reference, heterotrophs (>2.0 μm) were detected after removing phototrophic eukaryotes and detritus from the cytograms of FL1 and FSC (Fig. 3D) using logical gates. Thraustochytrids with FL3 and FL1 were detected in the cytogram of FL3 vs. FL1 (Fig. 3E). The reference polygon gate obtained for the Pooled-Mix sample, as shown in Fig. 3F, was then applied to specifically enumerate the thraustochytrid population in seawater.

**Fig. 4:**
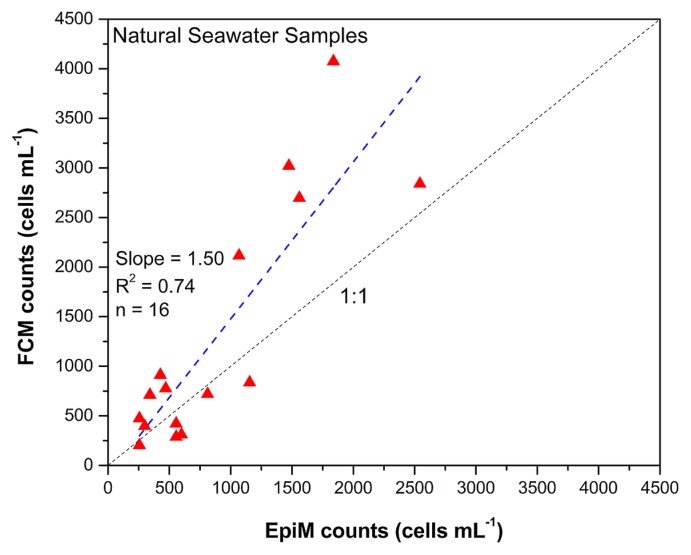
Comparison of epifluorescence microscopic and flow cytometry counts in natural seawater samples. Dotted (dark grey) and dashed (blue) lines indicate the 1:1 ratio and regression of data.

In order to validate our FCM protocol for its application to the enumeration of thraustochytrids in natural seawater, we compared the values of EpiM and FCM counts (assessed as described in the previous paragraph) of 30 natural seawater samples. The higher precision of FCM, defined as the low standard deviation of replicate sample analyses, than EpiM was achieved for most samples (Table S1). A regression of FCM and EpiM counts resulted in a line of slope 1.5 (Fig. 4) and showed higher FCM counts for most of the natural seawater samples than their EpiM counts. We found a fair agreement between the two methods (Fig. 4) even though the abundance of thraustochytrids by the FCM method was slightly higher than that of EpiM. Similarly higher abundance with the FCM method was found with culture samples in the present study. Nevertheless, our FCM results suggest the high precision of the method and more accurate enumeration of thraustochytrids in natural seawater.

### Abundance and biomass of thraustochytrids in coastal seawater

**Fig. 5:**
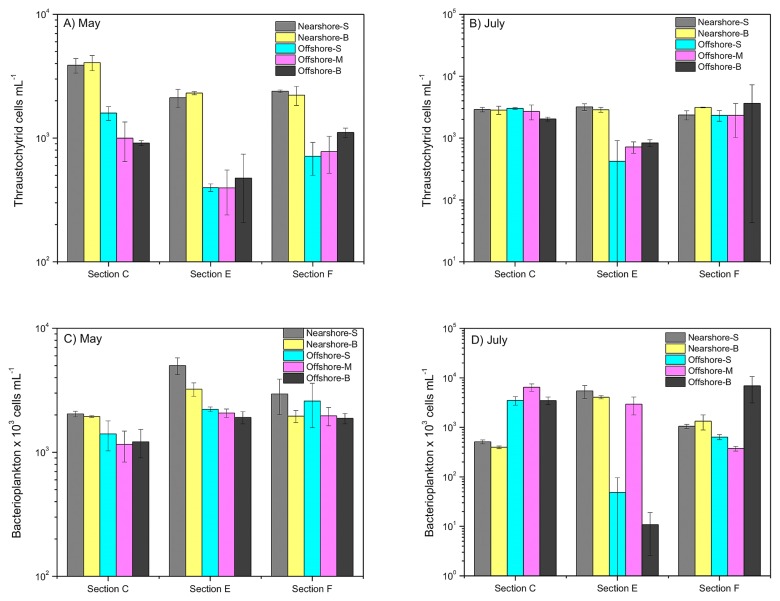
Abundance of thraustochytrids and bacterioplankton in natural seawater in nearshore stations (surface, subsurface, and bottom) and offshore stations (surface, subsurface, and bottom) along three sections: C, F, and E, respectively, in May and July. Thraustochytrid abundance was calculated from the flow cytometry counts of acriflavine-stained samples (A, B) and bacterioplankton abundance was calculated from the flow cytometry counts of SYBR-I Green-stained samples (C, D).

The abundance of thraustochytrids along the three sections in May (Fig. 5A) and July (Fig. 5B) 2014 was estimated and compared with that of bacterioplankton (Fig. 5C and D). The abundance of thraustochytrids ranged between 400 and 4,080 cells mL^−1^. The highest thraustochytrid abundance (4,080 cells mL^−1^) was detected at the bottom of the nearshore station along section C, while the lowest thraustochytrid abundance (400 cells mL^−1^) was at the surface and subsurface of the offshore station along section E. Abundance was the lowest and highest in May (Fig. 5A). The abundance of thraustochytrids across the three sections was distinct in May (*P*<0.05, Kruskal-Wallis test), exhibiting a significantly higher overall abundance in section C than in section E (*P*<0.05, Nemenyi tests). Notably, abundance in nearshore and offshore stations was significantly different in all sections in May (*P*<0.05, Nemenyi tests) and in section E in July. In contrast, the lowest bacterioplankton abundance (1.08×10^4^ cells mL^−1^) was detected at the bottom of the offshore station along section E, while the highest (6.87×10^6^ cells mL^−1^) was at the bottom of the offshore station along section F, both in July (Fig. 5D). Besides the significant difference observed in bacterioplankton abundance between nearshore and offshore stations in sections C and E in May (*P*<0.05, Nemenyi tests), their abundance in section C was significantly lower (*P*<0.05, Nemenyi tests) than that in other sections in May. Overall, May exhibited more variation in the abundance of thraustochytrids and bacterioplankton than July, suggesting more dynamic nutrient level changes in May in the seawaters of the Gulf of Bohai.

**Fig. 6:**
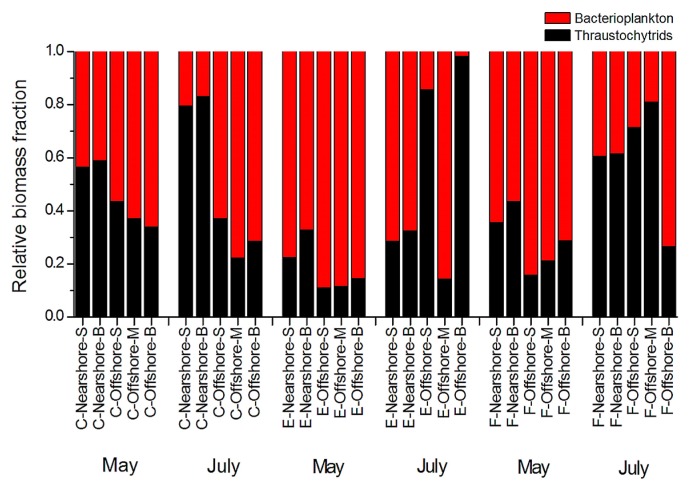
Relative biomass of thraustochytrids and bacterioplankton in the water column in nearshore stations (surface -S, subsurface -M, bottom -B) and offshore stations (surface -S, subsurface -M, bottom -B) along three sections: C, F, and E, respectively, in May and July.

The biomass of thraustochytrids ranged between 8.15 and 83.96 μg C L^−1^, contributing 10.9% to 98.1% of the total biomass of thraustochytrids and bacterioplankton (Fig. 6). The highest biomass (83.96 μg C L^−1^) was at the bottom of the nearshore station in section C, while the lowest (8.15 μg C L^−1^) was in the middle of the offshore station in section E, both in May. By comparison, the bacterioplankton biomass ranged between 0.33 and 194.57 μg C L^−1^. Of 30 seawater samples, two contained a higher biomass of thraustochytrids than that of bacterioplankton in May and eight in July (Fig. 6). Notably, we found the dominance of thraustochytrids over bacterioplankton in 1/3 of all samples. Our results clearly indicated the high relative biomass of thraustochytrids in July in the coastal waters of the Gulf of Bohai and our FCM protocol greatly assisted in revealing this seasonal pattern of the thraustochytrid biomass in these coastal waters.

### Factors affecting spatiotemporal abundance changes

**Fig. 7:**
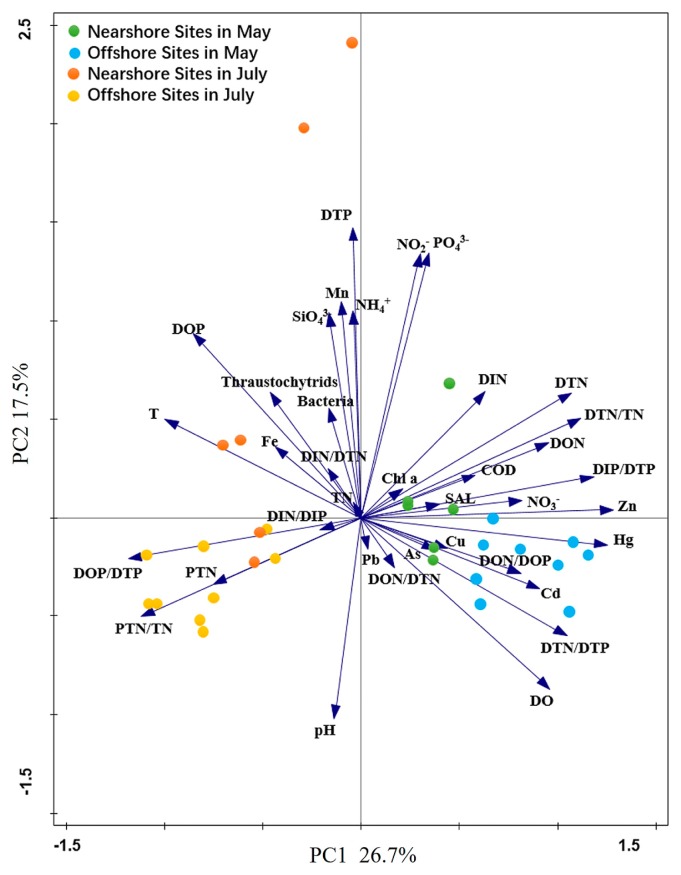
Ordination plot based on a Principal Component Analysis (PCA) of abundance and environmental datasets. The lines with arrowheads indicate the measured environmental variables and circles with different colors represent nearshore and offshore sampling stations in May and July.

The first (PC1) and second (PC2) principal components explained 26.7% and 17.5% of the total data variation (44.2%), respectively (Fig. 7). Based on the positions of the sample clusters on the ordination plot along PC1, it was evident that sample clusters across months (May and July) and shores (nearshore and offshore) possessed distinct environmental characteristics. PCA also indicated an overall greater temporal than spatial variation. The abundance of thraustochytrids positively correlated (*P*<0.05, Pearson’s test) with water temperature, Mn, Chlorophyll a, TP, and DOP, and negatively with DO (*P*<0.05) and DTN/DTP (*P*<0.01, Pearson’s test) (Table 1). These results indicated that the abundance of thraustochytrids in the coastal waters of the Gulf of Bohai was largely influenced by co-occurring physicochemical gradients.

## Discussion

### Detection and enumeration of thraustochytrids using FCM

Microscopy, quantitative PCR, and FCM are the most reliable cell enumeration techniques available to date. FCM is rapid, precise, and sorts cell types of interest, unlike microscopy and quantitative PCR. FCM is more than four-fold faster (3–5 min vs. 20 min per sample) and shows higher precision (<5% vs. >10% standard deviation) than microscopy (>20 min per sample) for characterizing microbial cells (44, 48, 49). The abundance and biomass of thraustochytrids have mostly been estimated using EpiM counts (4, 26, 30, 38). Although the EpiM technique has low error rates, it may be labor-intensive and subject to human error, particularly when many samples need to be analyzed in large-scale field studies. Thus, a replicate sample analysis is rarely feasible. We developed an FCM protocol to precisely estimate the abundance of thraustochytrids for large-scale studies. The results of the FCM protocol developed were similar to those of classical EpiM, but with better precision and more accuracy.

FCM provides useful information on the intrinsic characteristics of a microbial cell (3). The FSC parameter, which is the amount of light scattered at low angles, gives a direct relationship with the size of the particle, whereas the SSC parameter, the amount of light scattered at high angles, explains the complexity, granularity, and DNA/protein content (45). Even though light scattering is a function of the intrinsic characteristics of microbial cells, it is often difficult to distinguish among different microbial species based on this property alone. Therefore, cells may need to be stained with fluorescent dyes (43). In the present study, thraustochytrid cells were stained with the fluorescent dye acriflavine and the fluorescence emissions of cells were recorded in addition to light scattering parameters. Apart from minor shifts in the SSC, FL1, and FL3 parameter peaks for strains Mn16 and SW8, we did not observe more than one cluster of events in the FL1-SSC and FL1-FL3 cytograms of the culture samples. Thus, our FCM protocol did not allow for the unequivocal discrimination of thraustochytrid strains; we established a common and uniform region for the detection and enumeration of the thraustochytrid population in natural seawater. However, we noted distinct FCM signatures when the Mix sample at various growth stages was analyzed. The cytogram of Mix-24 h had distinct SSC and FL1 signatures (Fig. 2) that most likely resulted from the non-uniform cell size and DNA content of thraustochytrid cells in their exponential growth phase. Our FCM cytograms of mixed culture growth suggested an increasing population from the lag phase to the exponential and stationary phases. In a previous study on heterotrophic nanoplankton, similar population growth was evident from FCM cytograms (37). Hence, our FCM protocol incorporating the acriflavine dye staining of thraustochytrid cells allowed for the reliable differentiation of the mixed culture according to cell-cycle events and cellular DNA content. More importantly, the uniform region in the FL3-FL1 signature of the Pooled-Mix sample gated the events of all of the mixed culture samples over different growth stages, thereby constituting the characteristics of cell-cycle events. Hence, applying this FL1–FL3 region to gate events in natural seawater ensured that all cell-cycle events and cell states were included for enumeration. Therefore, proper gating is critical for revealing the intrinsic characteristics of microbial cells and also for accurate enumeration, particularly for seawater samples.

Seawater constitutes numerous cell types and cell states that make the detection and enumeration of target cell types difficult unless staining techniques are employed to discriminate each cell population. Staining becomes essential for most heterotrophic eukaryotes because they have little or no autofluorescence (37). Unfortunately, the most commonly used fluorescent compounds, such as SYTO 13, do not differentiate different microbial populations on a flow cytometer (37, 42, 46). We herein developed a new FCM protocol that employs the fluorogenic compound acriflavine, which selectively stains small unicellular eukaryotic cells (thraustochytrids) that contain sulfated polysaccharides in their cell wall and have nuclear DNA. We detected and enumerated thraustochytrids within seawater samples through a sequential analysis that distinguished them from phototrophic eukaryotes, heterotrophs, and detritus on the cytogram of FL1 vs. FL3 (Fig. 3). The results of the sequential analysis of natural seawater samples defined a uniform region on the cytogram for thraustochytrids and allowed the discrimination of thraustochytrids from other cell populations and particles. Our FCM protocol however did not exclude some cells, such as choanoflagellates, with similar fluorescent signal characteristics but different shapes (30). A small fraction of cells that appeared different from thraustochytrids by EpiM were indistinguishable by FCM. Apart from this, some particulates emitting totally brown or yellow fluorescence and some emitting FL1 in the cell wall and FL3 in the nucleus were also detected in the FL1–FL3 cytogram. The acriflavine staining method generally includes protozoan cysts and excludes thraustochytrid zoospores and young vegetative cells lacking cell walls (13, 30). Regardless of these intrinsic limitations of the acriflavine staining method, our FCM protocol markedly increased the efficiency of the analysis and realized the rapid enumeration of thraustochytrids in seawater. The FCM protocol developed may markedly reduce the time required to analyze large numbers of samples from large-scale field studies and also provide results with high precision and accuracy.

### Significance of high thraustochytrid abundance and biomass in coastal seawater

Small eukaryotes are important components of the microbial community in the ocean (20). Among these, thraustochytrids are known to thrive in a wide range of marine habitats ranging from tropical to temperate and cold waters (21, 36). The abundance and biomass of thraustochytrid protists have previously been described in only a few studies using direct detection techniques (4, 15, 19, 25). Using the FCM method, we found that the abundance and biomass of thraustochytrids often surpassed those of bacterioplankton in the coastal waters of the Gulf of Bohai, suggesting their importance in the carbon cycle and microbial food web. Differences in abundance and distribution among the three sections were consistent with previous findings, suggesting considerable temporal and spatial variations (35). In view of the large biomass of thraustochytrid protists in coastal waters and their positive correlations with chlorophyll *a* and DOP, we speculate that they may play a very important role in algal decomposition, similar to bacteria. Previous studies suggested that thraustochytrid protists, such as bacteria, primarily rely on phytoplankton carbon and thereby play a critical role in the marine microbial loop (21, 35).

Bacterioplankton and thraustochytrid protists form the base of the marine microbial food web, with the former being known for its utilization of the large pool of dissolved organic matter (31, 32). However, thraustochytrid protists are an often neglected component of the marine food chain (2), largely because of their acknowledged low abundance in contrast to bacterioplankton (13). The present study supports previous findings (1, 4, 15, 19) showing an occasionally higher biomass of thraustochytrid protists than of bacterioplankton (Fig. 6). The maximum abundance of thraustochytrids (4,080 cells mL^−1^) in the coastal waters of the Gulf of Bohai was markedly higher than the previously reported abundance of 1,757 cells mL^−1^ in the subtropical coastal waters of China. Our results also support coastal waters harboring a higher abundance of thraustochytrids than open oceans, *e.g.*, 670 cells mL^−1^ in the equatorial Indian Ocean (3) and 630 cell mL^−1^ in the Hawaiian waters (15). Similarly, the maximum abundance of bacterioplankton (6.87×10^6^ cells mL^−1^) in the coastal waters of the Gulf of Bohai was also higher than the previously reported abundance of 9.48×10^5^ cells mL^−1^ in the subtropical coastal waters of China. Our results strongly suggest a significant role for thraustochytrid protists in marine carbon cycling similar to bacteria.

Primary and secondary production and carbon biogeochemical processes in ecosystems are largely regulated by nutrient inputs from riverine and other anthropogenic activities through heterotrophic microbial communities. The high densities of thraustochytrids in the present study suggest that the coast of the Gulf of Bohai is a biologically productive region resulting from eutrophication because it has been strongly affected by terrestrial run-off. The high density of thraustochytrids in nearshore stations further indicates that the abundance of thraustochytrids is driven by terrestrial run-off (Fig. 5). This was further supported by the strong relationship between the physicochemical gradients and abundance of thraustochytrids (Table 1). In addition, each time period revealed different patterns for the abundance of bacterioplankton and thraustochytrids (Fig. 5), as reported elsewhere (4), suggesting that thraustochytrids and bacterioplankton either occupy distinct ecological niches in coastal waters or have different substrate preferences (35). The results presented herein are the first stemming from the FCM-based quantification of the abundance and biomass of thraustochytrids as well as their spatiotemporal distribution analysis. Our results further corroborate the important role of thraustochytrids in the marine microbial food web and suggest that more detailed investigations are needed in order to elucidate their role in coastal seawater.

In conclusion, the developed FCM method incorporating acriflavine staining is a very rapid and promising tool for the semi-automated enumeration of fungus-like protists. FCM is a practical and reliable method for detecting and enumerating thraustochytrids in culture and natural seawater samples. Our method anticipates the facilitation of large-scale studies on the abundance of thraustochytrids in the water column and is expected to realize a larger number of samples processed in a very short time span than the traditional EpiM method. Our case study on tracking the abundance and biomass of thraustochytrids using the FCM method revealed that thraustochytrids are a dynamic heterotrophic microbial group similar to bacterioplankton in coastal waters and suggests further research to investigate their literal ecological role.

## Supplementary Material



## Figures and Tables

**Table 1:** Pearson’s correlation between the abundance of thraustochytrids and environmental factors (only correlated factors are shown).

		T	DO	Mn	Chl a	TP	NO_3_^−^	DOP	DTN/DTP
Thraustochytrids	*r*	0.387	−0.439	0.370	0.413	0.582	−0.434	0.429	−0.527
	*p*	0.035	0.015	0.044	0.023	0.023	0.016	0.018	0.003

Note: T—water temperature; DO—dissolved oxygen; Chl a—Chlorophyll a; TP—total phosphorus; DOP—dissolved organic phosphorus; DTN—dissolved total nitrogen; *r*—Pearson’s correlation coefficient; *p*—significance level (two-tailed).
