# Supplementary material for: Flow Cytometry for Rapid Enumeration and Biomass Quantification of Thraustochytrids in Coastal Seawaters
Source: Microbes Environ. 2018 Jun 16;33(2):195–204. doi: 10.1264/jsme2.ME17162 (PMC6031391; doi:10.1264/jsme2.ME17162)
Supplement: Supplementary file 1 [file 33_195_s1.pdf]

Supplementary Information for

Flow Cytometry for Rapid Enumeration and Biomass Quantification of Thraustochytrids in Coastal Seawaters

Yingbo Duan[1,#], Biswarup Sen[1,#], Ningdong Xie[1], James S. Paterson[2], Wenmeng Ma[1], Zixi Chen[3], Guangyi Wang[1,*]

[#]These authors contributed equally to this work

[*]To whom correspondence should be addressed. Email: gywang@tju.edu.cn

Figure S1: Map of the sampling stations

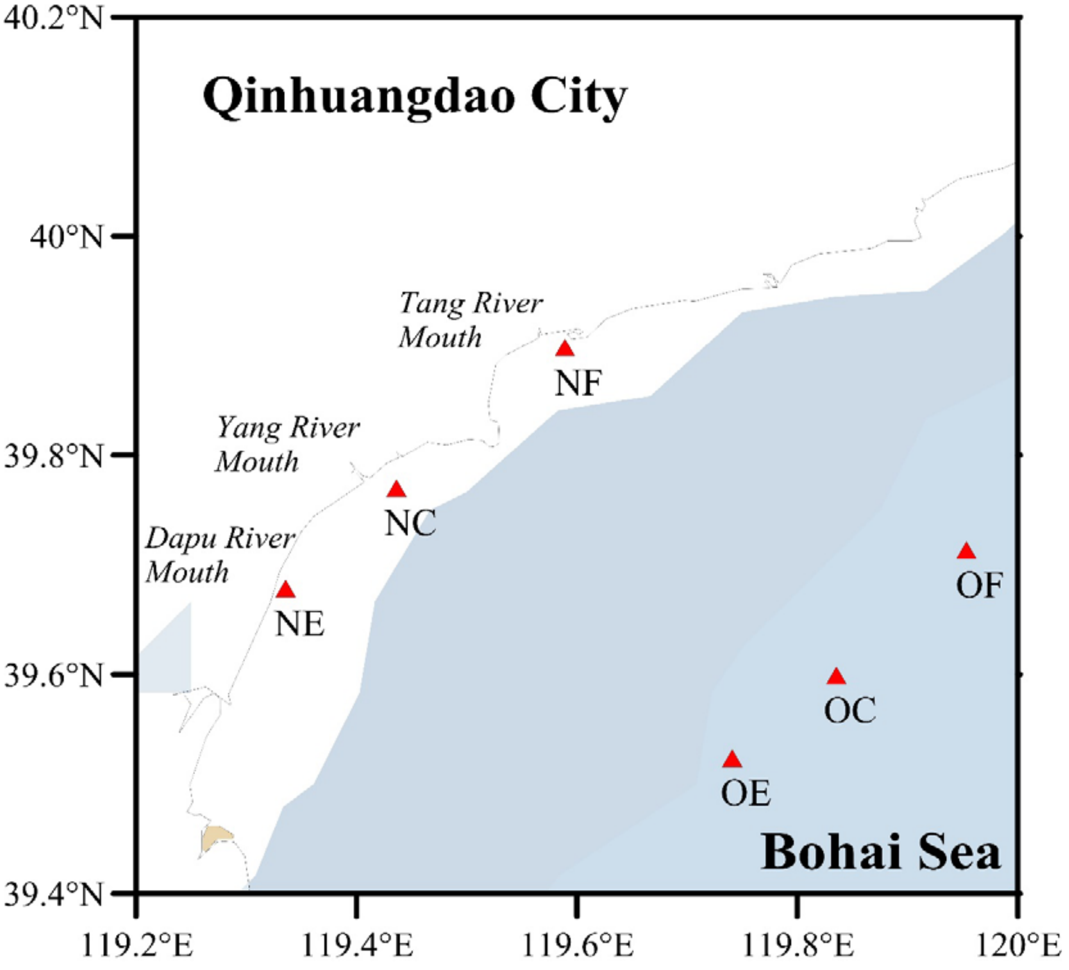

Figure S2: Epifluorescence photomicrograph of the sorted thraustochytrid cell in a natural seawater sample (scale bar is 5 µm).

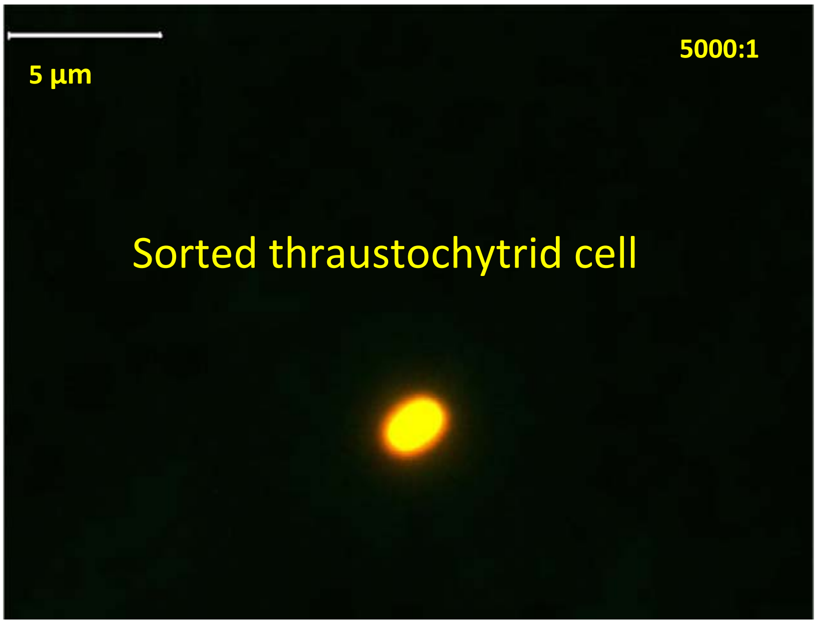

Figure S3: Histogram plot of the green fluorescence (FL1) of the five axenic cultures and their mixture sample ('Mix'). Beads are shown as the high fluorescence peaks. The peak area of 'Mix' sample is an average of all the peak areas of axenic cultures.

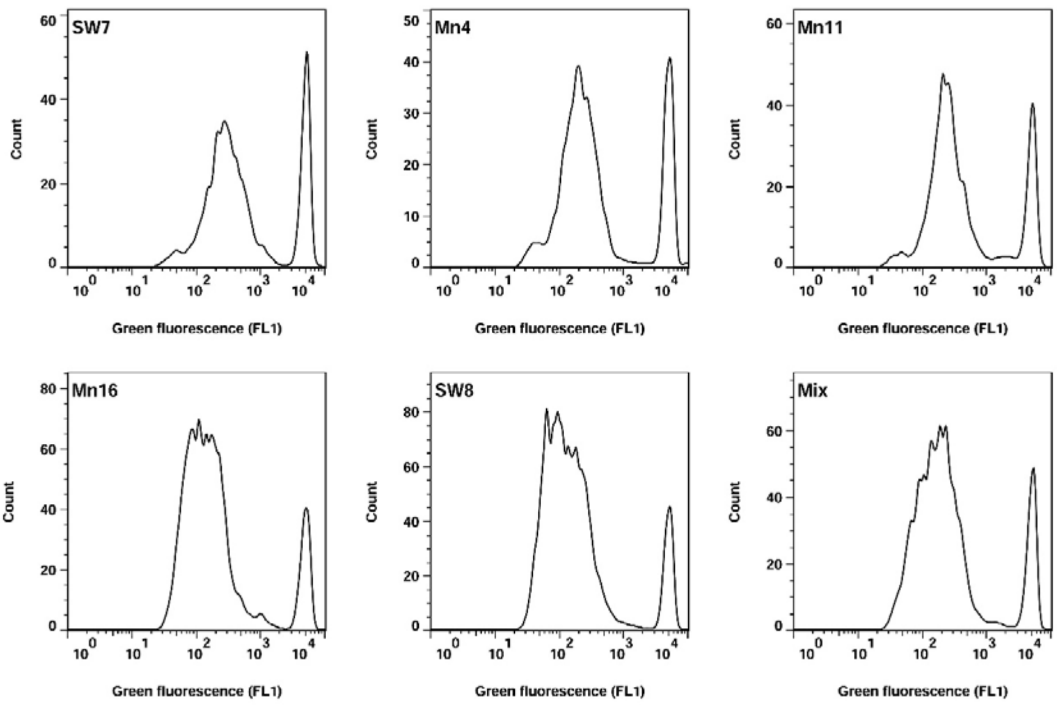

Table S1: Comparison of flow cytometry (FCM) and epifluorescence microscopy (EpiM) precision for seawater samples. Better FCM precision is determined by the low standard error (SD) of replicate sample analysis than that of EpiM.

**Samples**	**Month**	**Thraustochytrids abundance (10^3 cells/mL)**					**Thraustochytrids abundance (10^3 cells/mL)**			
		**FCM**					**EpiM**			
		Replicate-1	Replicate-2	Replicate-3	Mean	SD	Replicate-1	Replicate-2	Mean	SD
C11S	May	3.50	4.48	3.64	3.87	0.53	0.77	0.17	0.47	0.42
	July	3.05	3.02	2.61	2.89	0.25	1.03	0.90	0.96	0.09
C11B	May	4.73	3.68	3.81	4.08	0.57	0.60	3.08	1.84	1.75
	July	3.24	2.90	2.38	2.84	0.43	3.12	1.97	2.54	0.82
E01S	May	2.02	1.81	2.52	2.12	0.36	1.80	0.34	1.07	1.03
	July	3.59	2.80	3.17	3.18	0.40	1.75	0.73	1.24	0.73
E01B	May	2.38	2.31	2.24	2.31	0.07	0.51	0.68	0.60	0.12
	July	2.58	3.08	2.95	2.87	0.26	0.51	nd	nd	nd
F01S	May	2.33	2.45	2.39	2.39	0.06	0.77	0.34	0.56	0.30
	July	2.62	1.24	3.22	2.36	1.01	0.73	0.68	0.71	0.03
F01B	May	2.67	2.03	1.97	2.22	0.39	0.43	nd	nd	nd
	July	3.16	3.15	3.08	3.13	0.04	1.45	0.47	0.96	0.70
C21S	May	1.82	1.42	1.54	1.59	0.20	0.17	nd	nd	nd
	July	3.06	2.89	3.11	3.02	0.11	0.68	2.27	1.47	1.12
C21M	May	1.38	0.69	0.93	1.00	0.35	1.03	0.17	0.60	0.60
	July	3.18	3.05	1.86	2.70	0.73	0.81	2.31	1.56	1.06
C21B	May	0.92	0.88	0.94	0.91	0.04	0.51	0.34	0.43	0.12
	July	1.90	2.02	2.19	2.03	0.15	0.51	0.85	0.68	0.24
E10S	May	0.37	0.42	0.40	0.40	0.03	0.34	nd	nd	nd
	July	0.99	0.13	0.15	0.42	0.49	0.38	0.73	0.56	0.24
E10M	May	0.58	0.31	0.30	0.40	0.16	0.26	0.34	0.30	0.06
	July	0.62	0.65	0.89	0.72	0.15	1.20	0.43	0.81	0.54
E10B	May	0.44	0.76	0.23	0.47	0.27	0.26	nd	nd	nd
	July	0.81	0.75	0.95	0.84	0.10	1.37	0.94	1.15	0.30
F10S	May	0.92	0.71	0.50	0.71	0.21	0.34	nd	nd	nd
	July	2.59	1.80	2.57	2.32	0.45	0.81	0.94	0.88	0.09

F10M	May	1.05	0.54	0.74	0.78	0.26	0.26	0.68	0.47	0.30
	July	3.55	0.95	2.47	2.33	1.31	1.20	0.38	0.79	0.57
F10B	May	1.06	1.22	1.05	1.11	0.09	0.09	0.43	0.26	0.24
	July	0.82	7.70	2.42	3.65	3.60	1.33	1.71	1.52	0.27

*nd = not determined
